# Editorial: Neuroinformatics of large-scale brain modelling

**DOI:** 10.3389/fninf.2022.1043732

**Published:** 2022-10-11

**Authors:** John D. Griffiths, Kelly Shen, Padraig Gleeson

**Affiliations:** ^1^Krembil Centre for Neuroinformatics, Centre for Addiction and Mental Health, Toronto, ON, Canada; ^2^Department of Psychiatry, University of Toronto, Toronto, ON, Canada; ^3^Institute of Medical Sciences, University of Toronto, Toronto, ON, Canada; ^4^Institute for Neuroscience and Neurotechnology, Simon Fraser University, Burnaby, BC, Canada; ^5^Department of Neuroscience, Physiology, and Pharmacology, University College London, London, United Kingdom

**Keywords:** large-scale brain modeling, computational neuroscience, neuroinformatics, neuroimaging, simulators and models

A major focus in contemporary neuroscience research is the mapping and modeling of connectivity and activity dynamics in large-scale brain networks. As the resolution, coverage, and availability of neural data increase rapidly, neuroinformatics techniques are playing an increasingly important role in this scientific enterprise. *Large-scale brain modeling* is the methodologically-defined sub-field of computational neuroscience that is focused on simulations of either whole-brain activity at a coarse-grained (meso/macro) spatial scale, or activity in select neural subsystems at a fine-grained (micro) spatial scale and high level of detail. Neuroinformatics tools employed in large-scale brain modeling come in the form of software infrastructure, database resources, and practical implementations of mathematical and algorithmic techniques that facilitate these core research goals.

In many cases the neuroinformatics and architectural solutions developed as part of this work are in themselves of general methodological interest to researchers, but are often communicated secondarily to the principal neuroscientific research questions. This joint Frontiers in Neuroinformatics and Frontiers in Computational Neuroscience Research Topic was therefore conceived by the Editorial Team as a venue to highlight exciting recent developments in the field, as well as to demonstrate the broad range of innovative work taking place. It features a collection of 11 original research articles describing new advances in the neuroinformatics of large-scale brain modeling. These span a diverse range of computational methods and neuroscientific applications, from cell and microcircuit dynamics to macro-scale neuroanatomy and neuroimaging. In addition to the stand-alone value of the various individual contributions, we believe strongly that the shared focus on computational methodologies across the articles in this collection brings an important additional benefit—to facilitate dialogue, exposure, and cross-pollination across neuroscience sub-fields.

Two common themes across the included articles are (i) improving the scale, speed, accuracy, and resolution of modeling and data analysis pipelines, and (ii) improving connections between micro-meso-macro levels of analysis. We will discuss contributions from each of these themes in turn. Several of the featured papers describe new or improved simulator software. One example is the article by Panagiotou et al., which introduces a novel and high-performance neural simulator named EDEN (“Extensible Dynamics Engine for Networks”). By heavily basing EDEN around the model specification language NeuroML (Gleeson et al., 2010), EDEN achieves an impressive combination of flexibility and ease-of-use. More impressively however, the authors also demonstrate almost two orders-of-magnitude speed improvements as compared to the industry-standard tool NEURON (Hines and Carnevale, 1997) for simple single-computer usage, as well as seamless scaling over multiple CPUs and compute clusters with minimal effort and code modification. Improving computational scalability is also a major emphasis in the contributions of Florimbi et al. and Golosio et al., who describe impressive performance with new GPU-based architectures for modeling large-scale cerebellar networks (incorporating conductance-based neuronal models) and spiking network models, respectively. A common context where high-performance implementations are particularly needed and useful is in parameter space exploration and parameter optimization problems. On this topic, Yegenoglu et al. present a novel genetic algorithm-based approach, drawing on the concept of “learning to learn” (L2L), with worked examples for multiple simulators at multiple scales.

Complementing these contributions focused on the specification and execution of neural simulations, several articles in this collection address another major topic in the field of neuroinformatics, namely the systematic and efficient analysis of multimodal structural and functional brain data, at various spatial scales, as a critical first step in the development of large-scale computational models of brain activity. Moon et al. offer suggestions for improving the scalability of tractography analyses, now commonly used for reconstructing white matter fiber projections and anatomical connectivity patterns from individual human diffusion-weighted MRI scans. Their article is based around the interesting observation that probabilistic tractography reconstructions, which conventionally make use of thousands or millions of samples per seed location, can be reduced down to a handful of samples with comparable results in terms of identifiability and connectome matrix quality. Building on tools such as these, Frazier-Logue et al. describe a new improved neuroimaging pipeline for preprocessing brain connectivity using structural and functional MRI scans in preparation for whole-brain simulations with TheVirtualBrain library (https://thevirtualbrain.org; Sanz Leon et al., 2013).

Moving another level up, two articles in this collection explore the question of data organization *via* systematic ontologies. Gutierrez et al. describe a new tool for collaborative data-driven development of spiking network models, including structured management of the various entities used to specify physiological parameters and state variable dynamics, as well as code generation functionality that allows full specification of NEST-based network models (Gewaltig and Diesmann, 2007). At a slightly higher level of abstraction, Lazar et al. offer a novel “programmable logic” schema for describing the functional organization of the *Drosophila* brain, including a web application (“NeuroNLP++”) allowing natural language querying of published literature. These authors demonstrate usage of their new tool with examples exploring the functional logic of feedback loops in the *Drosophila* antennal lobe.

The second main theme in this Research Topic is, as noted, improving connections between micro, meso, and macro levels of analysis, which is represented by the articles from Siu et al., Layer et al., and Maith et al.. In the first of these, Maith et al. introduce a blood oxygenation-dependent (BOLD) “monitor” (i.e., empirical measurement process simulator) into the ANNarchy (Vitay et al., 2015) spiking neuron modeling library. This work can be seen as part of the major recent and growing trend in computational neuroscience toward “true” multiscale neural models that simultaneously capture experimentally observed patterns across several qualitatively different data types (D'Angelo and Jirsa, 2022). The contribution by Siu et al. also has a focus on modeling of hemodynamic activity patterns, in this case resting-state and stimulus-evoked fMRI activity patterns in anesthetized mice. Impressively, these authors combine theoretical analyses of bifurcation behavior in neural mass models of mouse cortex with neuroinformatics database-driven spatial variations in dynamical parameters to study resting-state and stimulus-driven activity patterns in (mouse) whole-brain functional MRI data. Finally, the paper by Layer et al. articulates a general approach for mean-field model derivation/reduction that is both theoretically powerful and practically useful, the latter in particular due to their development of the new open-source “Neuronal Network Mean-Field Toolbox” (NNMT) Python library (github.com/INM-6/nnmt). A particular strength of NNMT is its treatment of mean-field behavior for leaky integrate and fire model neuron models. The toolbox also provides functionality to estimate various properties of large neuronal networks, such as firing rates, power spectra, and dynamical stability in mean-field and linear response approximations, based entirely on well-developed mathematical theory and without the need for running computationally expensive numerical simulations.

We thank the authors for their excellent contributions to this Research Topic on the Neuroinformatics of Large-Scale Brain Modeling. Our hope and aim is that the collection assembled here provides an interesting and representative overview of the impressive recent work on this topic from around the world, and we look forward to continued discussions on new developments in this exciting field.

## Author contributions

All authors contributed equally to the editing of the Research Topic and conceptualization of the manuscript. JG wrote the first draft of the manuscript and all authors contributed to revisions and approved the submitted version.

## Funding

JG acknowledges research funding support from the Krembil Foundation, CAMH Discovery Fund, Labbatt Foundation, and Tri-Council UK-Canada AI Initiative. PG acknowledges research funding support from Wellcome (212941).

## Conflict of interest

The authors declare that the research was conducted in the absence of any commercial or financial relationships that could be construed as a potential conflict of interest.

## Publisher's note

All claims expressed in this article are solely those of the authors and do not necessarily represent those of their affiliated organizations, or those of the publisher, the editors and the reviewers. Any product that may be evaluated in this article, or claim that may be made by its manufacturer, is not guaranteed or endorsed by the publisher.
